# Aggressive unifocal bone Langerhans cell histiocytosis with soft tissue extension both responsive to radiotherapy: a case report

**DOI:** 10.1186/s13014-022-02108-0

**Published:** 2022-08-01

**Authors:** Wilmar Ghuijs, Paul G. Kemps, Marta E. Capala, Robert M. Verdijk, Astrid G. S. van Halteren, Robert J. P. van der Wal, Jan A. M. van Laar

**Affiliations:** 1 Department of Internal Medicine and Immunology, Erasmus MC University Medical Center Rotterdam, PO Box 2040, 3000 CA Rotterdam, The Netherlands; 2 Department of Pathology, Leiden University Medical Center, Leiden, The Netherlands; 3 Department of Radiotherapy, Erasmus MC University Medical Center Rotterdam, Rotterdam, The Netherlands; 4 Department of Pathology, Erasmus MC University Medical Center Rotterdam, Rotterdam, The Netherlands; 5 Princess Máxima Center for Pediatric Oncology, Utrecht, The Netherlands; 6 Department of Orthopaedics, Leiden University Medical Center, Leiden, The Netherlands

**Keywords:** Bone, Langerhans cell histiocytosis, LCH, BRAF, Radiotherapy

## Abstract

**Background:**

Langerhans cell histiocytosis (LCH) is a rare haematological neoplasm characterized by the accumulation of CD1a^+^, CD207/Langerin^+^ histiocytes within inflammatory lesions. LCH can involve any organ, but osteolytic bone lesions are most often encountered. Unifocal bone lesions may regress spontaneously after a thick needle biopsy has been taken.

**Case presentation:**

In this case report, we describe the initial presentation of a single *BRAF*^*V600E*^ mutated osteolytic LCH lesion in the left proximal humerus of a 46-year-old previously healthy woman. Despite multiple surgical interventions, she unexpectedly experienced progressive disease manifestation with significant soft tissue extension to the surrounding musculature, subcutis and epidermis. Because the disease manifestation remained loco-regional, radiotherapy (RT) (total dose of 20 Gy in 10 fractions) was initiated.

**Conclusion:**

The patient achieved a complete remission without any side effects. This case highlights that RT is a rational and relative mild local treatment option for patients with aggressive LCH affecting the bone and surrounding soft tissue.

## Background

Langerhans cell histiocytosis (LCH) is a rare haematological neoplasm characterized by the accumulation of CD1a^+^, CD207/Langerin^+^ histiocytes within inflammatory lesions. These neoplastic cells are the result of sporadic activating mutations in genes of the mitogen-activated protein kinase (MAPK) signaling pathway expressed by multipotent hematopoietic stem/progenitor cells or committed myeloid precursors [1]. LCH can affect any organ system, but most frequently occurs in the bones as osteolytic lesions. Therapeutic interventions depend on the number or site of involved organ systems [2]. In patients with a single bone lesion and no other organ involvement (so-called single-system unifocal bone LCH), taking a biopsy with or without subsequent surgical debulking (with or without intralesional steroids) followed by active monitoring is mostly applied. Although radiotherapy (RT) is not considered standard-of-care for osseous LCH [2], previous reports have suggested clinical benefit of this treatment modality in cases of uni- or multifocal (extra-)osseous single-system disease [3–5]. We present the rapid and sustained therapeutic efficacy of radiotherapy in an unusual case with progressive unifocal LCH of the proximal humerus involving both osseous and surrounding soft-tissue compartments.

## Case presentation

**Fig. 1:**
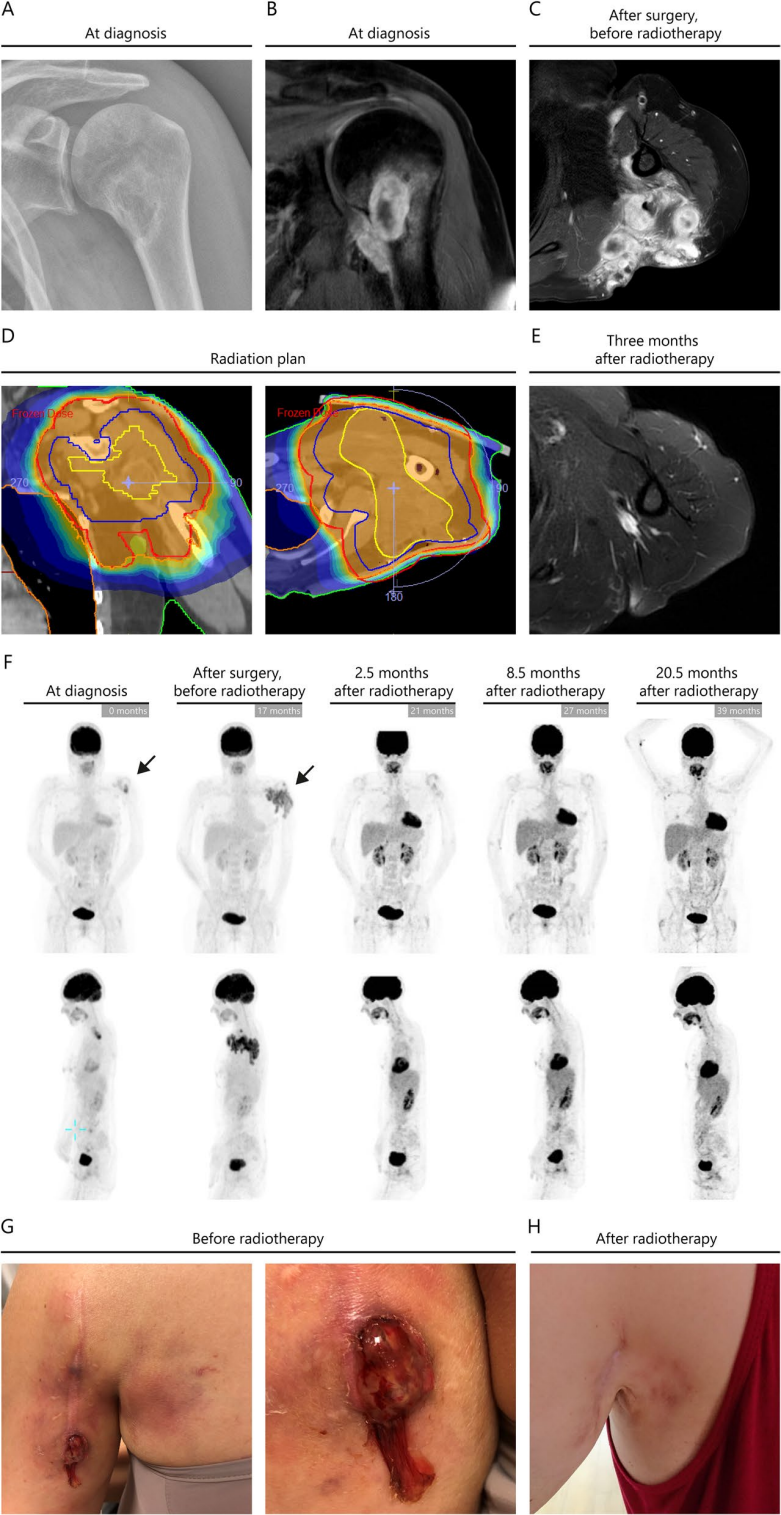
Coronal X-ray (**A**) and gadolinium-enhanced T1-weighted MRI (**B**) images demonstrating a unifocal lesion in the left proximal humeral diaphysis at time of diagnosis. After seventeen months and two surgical interventions, axial gadolinium-enhanced T1-weighted MRI demonstrated progressive disease with significant soft tissue extension (**C**). Radiation treatment plan of the humerus. The planning target volume (PTV) is indicated by the red line, the clinical target volume (CTV) by the blue line, and gross tumor volume (GTV) by the yellow line (**D**). Three months following RT, T2-weighted MRI demonstrates a full radiological response (**E**). Successive PET-CT images show strong FDG uptake in the bone and surrounding soft-tissues, with a remarkable decrease in both tumoral mass and FDG uptake after RT, compatible with a complete radiological response (**F**). Abscess-like soft tissue extension through the skin prior to RT (**G**). Clinical response at 9 months post RT (20 Gy in 10 fractions) showing marked improvement of skin and subcutis inflammation (**H**)

**Fig. 2:**
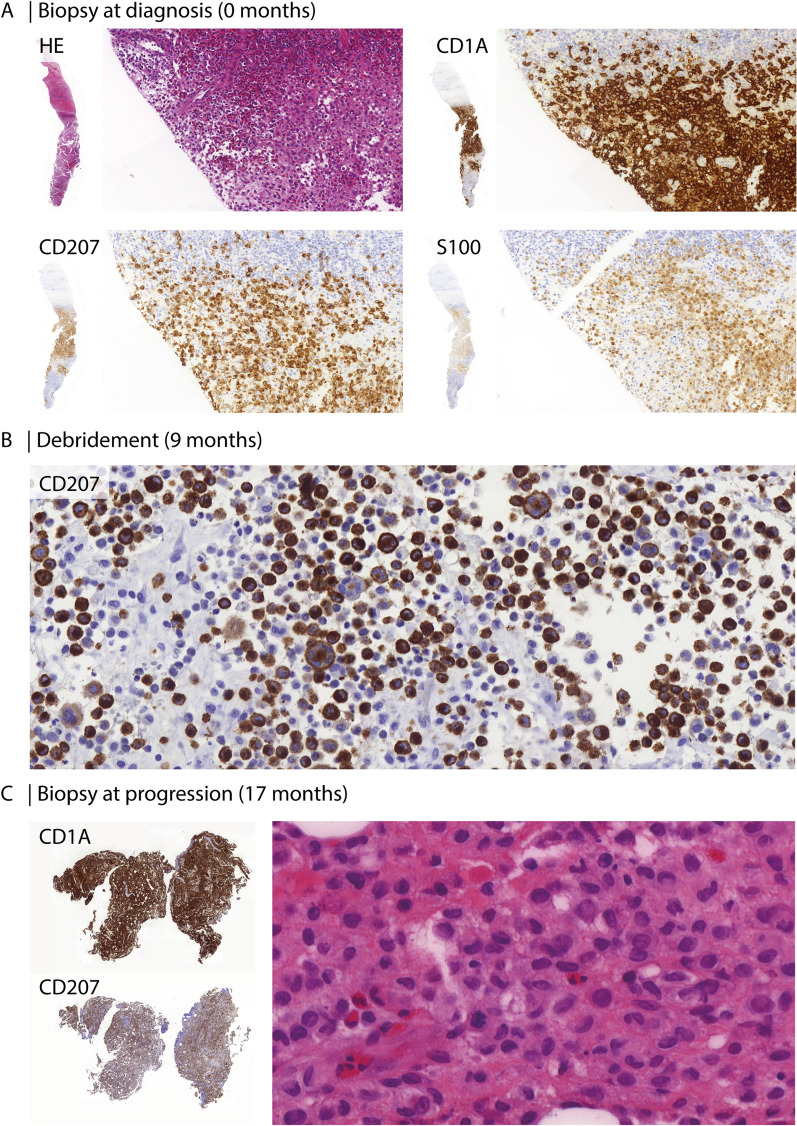
Hematoxylin and eosin (HE) and immunohistochemical stains of a thick needle biopsy demonstrate a dense infiltrate of CD1a and CD207 positive histiocytes with variable S100 expression (**A**). Immunohistochemical analysis of lesional tissue obtained at surgical debridement shows CD207 positive histiocytes and multinucleated giant cells (**B**). Histopathological analysis of a biopsy taken at time of disease progression demonstrates CD1a and CD207 positive histiocytes, indicative of active LCH, with frequent nuclear indentations and rare mitoses—one of which is shown (**C**)

A 46-year-old previously healthy woman was referred to the orthopedic surgeon because of progressive pain and dysmobility of her left shoulder since a few months. X-ray and magnetic resonance imaging (MRI) revealed an osteolytic lesion in the proximal humeral diaphysis of 2.3 × 2.0 × 4.0 cm (Fig. 1A, B). The major part of the lesion showed a sclerotic boundary, and there was high suspicion of glenohumeral joint involvement. In addition, cortical destruction was observed on the medial side. Given the radiologic aspect of the lesion and extensive reactive changes, subacute osteomyelitis with additional arthritis of the glenohumeral joint was initially suspected; malignant disease like chondrosarcoma was considered less likely. Histopathological analysis of a thick needle biopsy demonstrated, however, a dense infiltrate of S100, CD1a and CD207/Langerin positive histiocytes accompanied by many eosinophilic granulocytes (Fig. 2A), compatible with a diagnosis of LCH. Disease staging by fluorodeoxyglucose (FDG)-positron emission tomography (PET-CT) demonstrated no other active sites of disease. Surgical curettage and debridement was performed at five and nine months after initial presentation, respectively, due to progressive clinical complaints and radiological progression of the disease. Histopathological analyses performed on lesional tissue taken at each surgical intervention confirmed the presence of active LCH (Fig. 2B). Since surgical interventions often initiate a healing process in osteolytic LCH lesions [6], a watch-and-wait approach was initiated. Seven months after the debridement, the complaints returned and an MRI revealed a significant soft tissue mass surrounding the modestly increased humeral bone lesion, compatible with transformation into an “aggressive form” of unifocal bone LCH (Fig. 1C). PET-CT demonstrated strong FDG uptake in the lesion (Fig. 1F). A new biopsy ruled out malignant transformation or infectious complications and demonstrated, again, active LCH (Fig. 2C). Clinically, the patient also began to experience regional abscess-like soft tissue ulceration of the skin (Fig. 1G). Next-generation sequencing (NGS) performed on lesional DNA isolated from the second, third and fourth tissue sample from the affected humerus all demonstrated the same somatic *BRAF*^*V600E*^ mutation with a variant allele frequency ranging between 21–34% [7, 8] in the absence of copy number alterations. Using a more sensitive technique for detection of mutant *BRAF* alleles (droplet digital PCR) [9], *BRAF*^*V600E*^ was not detected in DNA extracted from myeloid or lymphoid cells isolated by flowcytometric sorting from a peripheral blood sample collected at the time of the fourth bone sample. Microbiological analysis of the bone and blood samples showed no signs of bacterial infection.

The unusual and aggressive clinical course necessitated intensified therapy. Both systemic (chemo)therapy and RT are rational treatment options for osseous LCH lesions with surrounding soft tissue extension [2–4]. Topical RT was preferred over systemic therapy because of the unifocal nature, expected better therapeutic efficacy, minimal risk of side effects, and low risk of LCH relapse generally associated with unifocal bone lesions, especially in adult patients. Based on previous reports [3, 4, 6], a total dose of 20 Gy in 10 fractions was administered, using a volumetric modulated arc therapy (VMAT) technique (Fig. 1D). This approach resulted in swift clinical improvement, with a notable reduction of pain already after 7 days. MRI and PET-CT imaging performed 2.5 months after the last RT dose showed a substantial decrease of both tumoral mass and FDG-uptake, indicating a significant radiological response (Fig. 1E, F). Follow-up MRI’s and PET-CT’s demonstrated complete radiological remission at 30 months after RT, without any new lesions. Complete cutaneous and soft tissue healing was observed at 9 months after RT (Fig. 1H).

## Discussion

Since decades, RT is sporadically applied in adults for the treatment of (particularly) osseous LCH lesions [4]. Rates of complete remission in adult LCH with uni- or multifocal osseous single-system disease range from 79–100% [6]. The disease course of the presented case is unusual since LCH bone lesions often respond well to surgery or can even resolve spontaneously [10]. However, our case had progressive localized disease with massive soft tissue extension. Both bone and soft tissue lesions responded swiftly to RT and our patient achieved complete remission without any side effects during the course of treatment, nor a relapse of the disease during the 30 months of follow-up thereafter. Thus, RT might be considered as a less toxic alternative for aggressive osseous LCH with extensive soft tissue involvement compared to systemic therapy, especially in adult patients with loco-regional disease manifestation.

NGS analysis performed on resected tissue collected at time of disease progression, and at 2 other time points in the disease course, consistently demonstrated the prototypical *BRAF*^*V600E*^ mutation [8]. Unfortunately, we could not verify that this mutation was already displayed by histiocytes present in the initial osteolytic lesion, as NGS analysis on DNA extracted from this decalcified specimen gave non-conclusive results. Since 2010, recurrent *BRAF*^*V600E*^ mutations have been found in approximately 50% of LCH cases [7]. An association between the oncogenic *BRAF*^*V600E*^ mutation and clinical parameters has been suggested in pediatric LCH [11], and the mutation also appeared to correlate with bone involvement in adults with LCH [12]. Given the aggressive clinical behavior in our patient, we specifically looked for additional genomic aberrations. Neither copy number variations nor additional cancer-associated driver mutations were found in any of the three tissue specimens, further opposing the possibility of LCH transformation into Langerhans cell sarcoma [13], which was also excluded based on the absence of major nuclear atypia and the presence of only sporadic mitoses.

## Conclusions

We describe a case where localized RT for an aggressive unifocal bone LCH lesion with striking soft tissue extension led to a robust and durable complete response. Although the bone lesion with aggressively behaving soft tissue extension displayed a targetable *BRAF*^*V600E*^ mutation, our case demonstrates that RT may be considered as a relative mild, yet effective, local treatment option for adult patients presenting with aggressive LCH involving the bone and/or soft tissues. Although RAF and MEK inhibitors are increasingly applied for refractory histiocytosis [14], reported toxicities [15, 16] in patients provide a rationale to choose RT above systemic therapy in selected cases.

## Data Availability

Data sharing is not applicable to this article as additional datasets were not generated.

## Abbreviations

LCH: Langerhans cell histiocytosis
RT: Radiotherapy
MAPK: Mitogen-activated protein kinase
MRI: Magnetic resonance imaging
FDG: Fluorodeoxyglucose
PET-CT: Positron emission tomography
NGS: Next-generation sequencing
